# Musculoskeletal manifestations in diabetic patients at a tertiary center

**DOI:** 10.3402/ljm.v7i0.19162

**Published:** 2012-10-29

**Authors:** Suzan M. Attar

**Affiliations:** Department of Internal Medicine, King Abdulaziz University, Jeddah, Saudi Arabia

**Keywords:** arthritis, diabetes mellitus, manifestations, musculoskeletal, rheumatological

## Abstract

**Objectives:**

Diabetes mellitus is a major public health problem worldwide. Most diabetic patients will develop functional disabilities due to multiple factors, including musculoskeletal (MSK) manifestations. The purpose of this study was to determine the frequency of MSK in diabetic patients and to examine the possible predictors for its development.

**Methods:**

We performed a cross-sectional study from June 1, 2010, to June 30, 2011, to evaluate MSK manifestations in adult diabetic patients at an outpatient clinic of King Abdulaziz University Hospital, Jeddah, Saudi Arabia. Baseline variables were examined to determine predictors for the development of MSK complications. Analyses were carried out using the Statistical Package for Social sciences.

**Results:**

We included 252 diabetic patients; 45 (17.9%) had MSK manifestations. Of these 45 patients, 41 (91.1%) had type 2 diabetes. The most common manifestations were carpal tunnel syndrome (*n*=17, 6.7%), shoulder adhesive capsulitis (*n*=17, 6.7%), and diabetic amyotrophy (*n*=12, 4.8%). A significant association was found between the development of MSK manifestations and manual labor, overweight, and vascular complications. On logistic regression analysis, the presence of vascular complications in general (B-coefficient=1.27, odds ratio=3.57, *P*<0.05, 95% confidence interval=1.31–9.78), and retinopathy in particular (B-coefficient=1.17, odds ratio=3.21, *P*<0.05, 95% confidence interval=1.47–7.02) can predict the development of MSK manifestations in about 82% of the cases.

**Conclusion:**

Musculoskeletal manifestations are under recognized in adult diabetic patients, occurring in 18% of the cases. Physicians should consider examining the periarticular region of the joints in the hands and shoulders whenever a diabetic patient presents with MSK symptoms.

Musculoskeletal (MSK) complications of diabetes mellitus (DM) are the most common endocrine arthropathies. These have been generally under-recognized and poorly treated compared with other complications, such as neuropathy, retinopathy, and nephropathy. These manifestations, which are some of the causes of chronic disability, involve not only the joints, but also the bones and the soft tissues. In 2004, the National Health Interview Survey determined that 58% of diabetic patients would have functional disability (1). The percentage of diabetic patients with functional disability will increase as the number of diabetic patients increases, and hence constitute a major public health problem. Recent data show that the prevalence of MSK manifestations in the hands and shoulders in patients with type 1 or type 2 diabetes is 30% (2). These manifestations are closely linked to age (3), prolonged disease duration (4, 5), and vascular complications in the form of retinopathy (6).

In Arab countries, there is a paucity of reports that describe MSK disabilities in diabetic patients. No previous studies had been conducted to assess the prevalence of MSK manifestations in diabetic patients or to evaluate the predisposing factors. Thus, the aim of this study was to evaluate the frequency of MSK manifestations in adult diabetic patients visiting a tertiary center in Jeddah, Saudi Arabia, and to identify the clinical and biochemical risk factors for the development of these complications.

## Methods

We performed a cross-sectional study to evaluate MSK complications in adult diabetic patients at the Endocrinology Clinic of King Abdulaziz University Hospital, Jeddah, Saudi Arabia, between June 2010 and June 2011. King Abdulaziz University Hospital is a tertiary center known to provide health care to a multinational population of mixed socio-economic status. Consent was obtained from the patients prior to their inclusion in the study. The study was approved by the Biomedical Ethical Research Committee of the Faculty of Medicine of King Abdulaziz University.

A rheumatologist examined all adult patients with type 1 and 2 diabetes for MSK manifestations of diabetes. Patients were included provided they had a history of DM for at least 2 years, diagnosed according to the World Health Organization (WHO) as a fasting plasma glucose level of ≥126 mg/dL (7.0 mmol/l) (7). Patients with rheumatoid arthritis, osteoarthritis and osteoporosis were excluded. Osteoarthritis was considered if the patients had the classic changes of osteoarthritis in their hands, including Heberden's and Bouchard's nodes.

For all patients included in the study, we recorded the following information: demographic features, including age, gender, nationality, and occupation, especially manual labor, smoking habits, and the body mass index (BMI), which was calculated as weight in kilograms divided by height in meters squared. We considered overweight subjects with a BMI between 25 and 29.9 and obesity with BMI>30 kg/m^2^ as per WHO 2000 classification for BMI (8). We obtained the following clinical information: duration of diabetes (in years); type of diabetes (type 1 or 2); disease control; complications due to diabetes; retinopathy (confirmed by an ophthalmologist or patient undergoing laser treatment); neuropathy (defined as mononeuropathy, peripheral, or autonomic neuropathy); nephropathy (defined as microalbuminuria, proteinuria, or renal failure); coronary artery disease (CAD); and cerebrovascular accident (CVA) (9). We also recorded the hemoglobin A1C (HbA1c) levels of the patients, measured by immunoassay, using the Cobas Integra 800 automated analyzer (Roche Diagnostics, Manheim, Germany) (10). We calculated the mean HbA1c level from results obtained at the most recent visit and those obtained at the last three, over the previous year, to provide an integrated index of glycemic control during follow up. Poor glycemic control was considered when HbA1c levels were >8.0%.

MSK manifestations in patients with diabetes were divided into three categories: (1) disorders that represent intrinsic complications of diabetes, such as diabetic cheiroarthropathy and diabetic muscle infarction; (2) disorders with an increased incidence among diabetic patients, such as Dupuytren's disease, shoulder capsulitis, neuropathic arthropathy, flexor tenosynovitis, de Quervain's tenosynovitis, reflex sympathetic dystrophy (RSD), diabetic amyotrophy, diffuse idiopathic skeletal hyperostosis, diabetic osteolysis, septic arthritis, osteomyelitis; and (3) disorders that have been possibly linked to diabetes, such as carpal tunnel syndrome (CTS) and crystal arthropathy (11–13).

A systematic method was used to evaluate the patients. First, the hands were examined, followed by the shoulders, then spine, and finally the lower limbs. The following definitions were used to identify all of the manifestations:

1. Diabetic cheiroarthropathy or limited joint mobility was evaluated by the patient ‘prayer sign’ in which the patient was asked to approximate the palmar surfaces of their interphalengeal joints, with the fingers fanned and the wrist maximally extended. If they were unable to do so, the test was considered positive (13).
2. Diabetic sclerodactyly was defined as thickening of the skin on the dorsal aspect of the hand in association with limited joint mobility in the absence of Raynaud phenomenon, calcinosis, and telangiectasia.
3. CTS was defined as weakness or pain of the hand, evidence of thenar atrophy, or nocturnal paresthesia of the thumb, index, and long fingers, with or without a positive Tinel's or Phalen's sign. CTS was excluded if other causes, such as thyroid disease, acromegaly, or C5/C6 radiculopathy were suspected. We also considered a history of surgery as evidence of the disease.
4. Flexor tenosynovitis or stenosing tenosynovitis or trigger finger was diagnosed by palpating a nodule or thickened flexor tendon with locking phenomenon during finger flexion or extension.
5. The diagnosis of Dupuytren's contracture was based on one of the following features: a palmar or digital nodule; tethering of palmar or digital skin; a pretendinous band and a digital flexion contracture, palpable thickening of the palmar fascia, with a flexor deformity of the second, third, fourth, or fifth fingers.
6. RSD was defined as unilateral, localized, or diffused pain associated with swelling or trophic changes and vasomotor disturbance with impaired mobility of the affected limb.
7. Diabetic osteolysis was characterized by osteoporosis of the proximal phalanges in the hands and feet, documented by X-ray radiographs.
8. Patients with unilateral shoulder pain for at least 1 month, an inability to lie on the affected shoulder, and restricted active and passive shoulder joint movement of less than 50% in at least three planes were diagnosed as frozen shoulder or adhesive capsulitis.
9. Diffuse idiopathic skeletal hyperostosis syndrome was diagnosed based on the classification criteria set by Resnick and Niwayama, which requires radiographically recognized bridges connecting at least four contiguous vertebrae of the thoracic spine, with preservation of the intervertebral disk space and absence of apophyseal joints or sacroiliac inflammatory changes (12–14). Only those with back pain had an X-ray of the spine.
10. Charcot joint or neuropathic arthropathy was defined as painless swelling and deformity at the weight-bearing joints and the classical finding of articular surface destruction, dislocation, disorganization, and increased density of the involved joint on X-ray radiographs.
11. Diabetic amyotrophy was defined as wasting of the proximal upper or lower extremity muscles or the paraspinal muscles, preceded by severe pain and dyesthesia of the involved part.
12. Diabetic muscle infarction was defined as a palpable painful mass with swelling and induration of the surrounding tissue without systemic symptoms, in addition to evidence of edema in the muscle on magnetic resonance imaging. A history of surgery for was also considered as evidence of the disease (12–14).

## Statistical analysis

Data analysis was carried out using the Statistical Package for Social Sciences (SPSS Inc., Chicago, IL, USA), version 16. Mean±standard deviation (SD) was calculated for quantitative data and percentage for categorical variables. Student's *t*-test was used for comparing the means of continuous variables. Numbers and percentages were compared by Chi-square test and Mantel–Haenszel test if needed. The predictive value of MSK manifestations for the various co-variables was calculated using multivariate logistic regression analysis, with a confidence interval of 95%. Results were considered significant if the *P-*value was <0.05.

## Results

A total of 252 diabetic patients were examined during the study period. The mean age of the patients was 56.71±14 years. Eighty patients (31.7%) were Saudis. Non-Saudis constituted 68.3% of the study population and originated from different ethnic backgrounds. Fifty (19.8%) patients did manual labor. Twenty-six patients (10.3%) were smokers.

The mean duration of diabetes was 11.21±8.5 years. Type 1 diabetes was diagnosed in 36 patients (14.3%); 215 patients (85.3%) had type 2 diabetes. Only 31 patients (12.3%) had controlled diabetes, with mean HbA1c level of 8.6±2.3 mmol/L (range, 1–17 mmol/L). Sixty-nine patients (27.4%) were overweight, while 128 patients (50.8%) were obese. Vascular complications were observed in 168 patients (66.7%) in the form of retinopathy in 38 (15.1%) patients, neuropathy in 120 patients (47.6%), nephropathy in 53 (21.0%) patients, CAD in 75 patients (29.8%) and CVA in 21patients (8.3%).

MSK manifestations were diagnosed in 45 (17.9%) of 252 patients. MSK disorders were more common in patients with type 2 diabetes (*n*=41, 91.1%) than in those with type 1 diabetes (*n*=4, 8.9%). Almost half (48.9%) of the patients with MSK disorders had more than one manifestation. The manifestations were CTS and shoulder capsulitis in 17 cases (6.7%) each, diabetic amyotrophy in 12 patients (4.8%), flexor tenosynovitis in 11 patients (4.4%), diabetic cheiroarthropathy in 8 patients (3.2%), crystal arthropathy in 7 patients (2.7%) (5 patients had gout, while 2 patients had pseudogout), diabetic sclerodactyly in 5 patients (2.0%), and plantar fasciitis in 3 patients (1.2%). Dupuytren's disease, muscle infarction, neuropathic arthropathy, diffuse idiopathic skeletal hyperostosis, de Quervain's tenosynovitis, and osteomyelitis were found in one case each (0.4%). None of the patients had digital osteolysis, RSD, or septic arthritis (Table 1).

**Table 1:** Prevalence of musculoskeletal disorders among studied diabetic patients^a^

MSK manifestations	Number	%
Overall prevalence	45	17.8
Carpal tunnel syndrome	17	6.7
Shoulder adhesive capsulitis	17	6.7
Diabetic amyotrophy	12	4.8
Flexor tenosynovitis	11	4.4
Diabetic cheiroarthropathy	8	3.2
Crystal arthropathy	7	2.7
Diabetic sclerodactyly	5	2.0
Plantar fasciitis	3	1.2
Dupuytren's disease	1	0.4
Muscle infarction	1	0.4
Charcot joint	1	0.4
Diffuse idiopathic skeletal hyperosteosis	1	0.4
De Quervain's tenosynovitis	1	0.4
Osteomyelitis	1	0.4
Diabetic osteolysis	0	0.0
Reflex sympathetic dystrophy	0	0.0
Septic arthritis	0	0.0

MSK, musculoskeletal manifestation.

a Several features may co-exist.

Table 2 shows the association between the clinical variables of diabetes control and the MSK manifestations of DM. There was a significant association between the development of MSK manifestations and manual labor, overweight, and vascular complications. By logistic regression analysis, vascular complications (B-coefficient=1.27, odds ratio [OR]=3.57, *P*<0.05, 95% confidence interval [CI]=1.31–9.78) and retinopathy, in particular (B-coefficient=1.17, OR=3.21, *P*<0.05, 95%CI=1.47–7.02), were predictors of MSK complications in about 80% of the cases (Table 3).

**Table 2:** Characteristics of patients with and without musculoskeletal manifestations and the association between 45 patients with musculoskeletal manifestations and clinical variables^a^

Predictor	With MSK, *n* =45 (%)	Without MSK, *n* =207	*χ* ^2^	*P*	OR	95% CI
Age in years (mean±SD)	59.63±9.7	56.10±14.7		0.13^b^		
Gender						
Female (*n*=147)	31 (21.1)	116 (78.9)	2.33	0.127^a^	1.095	(0.98–1.22)
Male (*n*=105)	14 (13.3)	91 (86.7)			RC	
Nationality						
Saudi (*n*=80)	17 (21.3)	63 (78.8)	0.92	0.338^a^	1.31	(0.76–2.24)
Non-Saudi (*n*=172)	28 (16)	144 (84)			RC	
Manual labor						
Yes (*n*=50)	4 (8)	46 (92)	4.1	0.042^a^^,^ b	0.39	(0.15–1.05)
No (*n*=202)	41 (20.3)	161 (79.7)			1.2	(1.04–1.3)
Smoking (*n*=26)	3 (11.5)	23 (88.5)	0.8	0.37^b^	0.62	(0.21–1.86)
Duration of diabetes (mean±SD)	12.07±6.84	11.01±11.02		0.001^a^^,^ c		
Disease not controlled (*n*=220)	41 (13)	27 (87)	0.61	0.44^b^	1.07	(0.92–1.24)
Type of diabetes						
Type 1 (*n*=37)	5 (14)	32 (86)	1.303	0.245^b^	0.55	(0.18–1.59)
Type 2 (*n*=215)	41 (19)	174 (81)	1.55	0.3	1.2	(0.96–1.25)
Weight						
Underweight (*n*=11)	2 (18)	9 (82)	0.03	0.56^b^	1.15	(0.23–5.64)
Normal (*n*=44)	13 (30)	31 (70)			RC	
Overweight (*n*=69)	5 (10)	64 (90)	7.3	0.007^a^^,^ b	0.332	(0.14–0.81)
Obesity (*n*=128)	25 (19)	103 (81)	0.49	0.25	1.26	(0.66–2.41)
Hemoglobin A1c level	8.5±1.94	8.6±2.3		0.8		
Complications						
Yes (*n*=168)	40 (24)	128 (76)	12.17	0.001^a^^,^ b	4	(1.64–9.76)
No (*n*=84)	5 (6)	79 (94)			RC	(0.077–0.54)
Retinopathy						
Yes (*n*=38)	16 (42)	22 (58)	17.94	0.001^a^^,^ b	3.1	(1.88–5.14)
No (*n*=214)	29 (14)	185 (86)			RC	
Nephropathy						
Yes (*n*=53)	14 (26)	39 (74)	3.3	0.07^b^	1.7	(0.97–2.93)
No (*n*=199)	32 (16)	167 (84)			RC	
Neuropathy						
Yes (*n*=120)	30 (25)	90 (75)	7.97	0.005^a^^,^ b	2.2	(1.25–3.88)
No (*n*=132)	15 (11)	117 (89)			RC	
CAD						
Yes (*n*=75)	21 (28)	54 (72)	7.5	0.006^a^^,^ b	2.1	(1.23–3.47)
No (*n*=177)	24 (14)	153 (86)			RC	
CVA						
Yes (*n*=21)	6 (29)	19 (71)	1.79	0.181^b^	1.69	(0.81–3.53)
No (*n*=231)	39 (17)	192 (83)			RC	

CAD, coronary artery disease; CI, confident interval; CVA, cerebrovascular accident; MSK, musculoskeletal; OR, odds ratio, RC, Reference category.

Data were presented as frequencies (percentages) unless otherwise stated.

a Significant association, *P*<0.05.

*P* was calculated by

b Chi-square test,

c Mann–Whitney U test.

**Table 3:** Determination of the most significant predictors of musculoskeletal manifestation using multivariate logistic regression analysis

Predictors	B-coefficient	*P*	Exact risk	95% CI
(A) Significant predictors				
Vascular complications	1.27	**0.001** *	3.57	(1.31–9.78)
Retinopathy	1.17	**0.001** *	3.21	(1.47–7.02)

* Significant association, *P*<0.05.

There was a significant association between certain manifestations and predictors: CTS and retinopathy, neuropathy and CAD, shoulder capsulitis and retinopathy and CAD, flexor tenosynovitis with retinopathy and neuropathy, diabetic cheiroarthropathy and diabetic sclerodactyly with retinopathy and CAD. Also, an association was observed between one manifestation and another: Shoulder adhesive capsulitis with CTS (*P*<0.05), flexor tenosynovitis (*P*<0.007), and limited joint mobility (*P*<0.001). Table 4 summarizes the MSK manifestations in relation to weight and vascular complications of DM by Chi-square testing.

**Table 4:** Musculoskeletal manifestations and their relation to weight and complications of diabetes mellitus^a^

Predictor Musculoskeletal manifestation, *n*=45 (%)	Weight		Complication			
	
	Overweight (*n*=69)	Obesity (*n*=128)	Retino (*n*=38)	Nephro (*n*=53)	Neuro (*n*=120)	CAD (*n*=75)
Carpal tunnel syndrome (*n*=17)	2 (12)	9 (53)	8 (47)* a	6 (35)^a^	12 (71)* b	9 (53)* b
Shoulder capsulitis (*n*=17)	3 (18)^a^	8 (47)^a^	10 (59)* a	8 (47)^a^	11 (65)^b^	10 (59)* b
Diabetic amyotrophy (*n*=12)	2 (16)	6 (50)	3 (25)	2 (17)	7 (58)	3 (25)
Flexor tenosynovitis (*n*=11)	0	5 (45)^b^	6 (54)* a	2 (18)^a^	10 (91)* b	3 (27)^a^
Diabetic cheiroarthropathy (*n*=8)	4 (50)	2 (25)	5 (63)*	3 (38)	4 (50)	7 (88)*
Crystal arthropathy (*n*=7)	1 (14)	3 (43)	1 (14)	3 (43)	4 (57)	2 (29)
Diabetic sclerodactyly (*n*=5)	2 (40)^a^	2 (40)^a^	5 (100)*	2 (40)	3 (60)	4 (80)*
Plantar fasciitis (*n*=3)	0	3 (100)	1 (33)	1 (33)	3 (100)	1 (33)

Nephro, nephrology; Neuro, neurology; Retino, retinopathy; CAD, coronary artery disease.

Data are presented as frequency (percentages) unless otherwise stated. Multiple features were present.

Calculated by

a Fisher's exact test and

b Chi-square test.

* Significant association, *P*<0.05.

## Discussion

Our study showed three important findings. First, the frequency of MSK manifestations in DM was 17.9%. Second, the most common MSK manifestations were CTS and adhesive capsulitis (6.7% in each case). Third, there was a significant association between vascular complications and the development of MSK manifestations.

It is estimated that more than 50% of diabetic patients will suffer from chronic disability (1). Some factors that contribute to chronic disability in diabetic patients include vascular complications, in addition to predisposing conditions, such as obesity and low physical activity. It was reported that patients with type 2 diabetes had greater impairments in mobility and more difficulties performing basic activities of daily living (ADL) than similarly aged non-diabetic persons (15). This leads to loss of independence, and it may predict future hospitalization, institutionalization, and death (16).

Many studies have evaluated MSK manifestations in diabetic patients, but most assessed only an individual component, especially MSK involvement of the hands and shoulders (2–4). Only few studies evaluated the whole MSK system, including the limbs and back. Amongst these was a pilot study on 208 patients with type 2 diabetes in which MSK complications, excluding osteoarthritis, were detected in 38% of the cases (3). A higher prevalence (58%) was reported by other authors in a cohort of 80 patients with type 2 diabetes (17). The lower prevalence of MSK manifestations in our cohort could be related to the fact that we excluded osteoarthritis, which was a common finding in our study population.

The most frequent MSK complication in this study was CTS, observed in (6.7%) of our patients. In the literature, CTS is observed in up to 20% of patients with diabetes (18). Similar to CTS, adhesive capsulitis was observed in 6.7% of our patients; however, this was less than the 25% reported from a British cohort. (19) Adhesive capsulitis is a chronic disabling condition associated with pain, requiring long-term treatment in the form of physiotherapy and repeated injections. Unfortunately, the treatment is more prolonged in diabetic patients, and surgical intervention may be required if the condition is not treated early (20).

There is strong evidence that the development of one complication predisposes to the development of another. We found a significant association between shoulder adhesive capsulitis and other MSK complications of diabetes, notably CTS, flexor tenosynovitis, and limited joint mobility. Similarly, a Japanese group examined 302 diabetic patients in a case-control study and demonstrated a significant association between different types of complications, especially flexor tenosynovitis and limited joint mobility (20).

Over the past years, it has been shown that these strong predictors predisposed to the development of MSK complications in diabetic patients, (3, 19, 21–25) of which the most important is blood glucose control. In our study, there was no association between blood glucose control and MSK manifestations although we calculated the mean HbA1c level from results obtained during the last three visits, as a single HbA1c level does not correlate with tissue levels of advanced glycosylation end products (23). This is in line with the findings of Thomas et al. (24) but contradicts the results obtained in a British cohort that demonstrated a strong association between MSK manifestations and poor blood glucose control (19).

Vascular complications are another important predisposing factor. We found a significant association between MSK manifestations and vascular complications. Ardic et al. (3) demonstrated that hand and shoulder syndrome was most likely associated with retinopathy but not nephropathy or neuropathy, in line with to our findings. Although we detected a mathematical correlation between MSK complications and vascular complications, retinopathy was the most significant predictor, with patients having at least an 85% chance of developing MSK complications.

Occupations that involved manual labor increased the risk of hand complications in our patients. This finding is in line with those reported by other authors. (22, 25) The risk of flexor tenosynovitis and CTS was higher in our patients with peripheral neuropathy. This could be attributed to the loss of sensation in addition to the fact that the patients presented late when surgery was mandatory.

At present, there are established guidelines for the follow up of type 2 diabetic patients, which are aimed at detecting microvascular complications, such as retinopathy, neuropathy, and nephropathy (26). However, none has been established to guide clinicians to follow up these patients for the detection of MSK complications. Clinicians should therefore be aware of the possible MSK complications of diabetes to intervene and provide the best care for patients presenting with rheumatologic disorders. Asking patients about their symptoms and monitoring for signs of MSK complications can be an important component of diabetes care.

## Conclusion

Although not commonly recognized as a complication of diabetes, MSK manifestations are present in about 18% of diabetic patients. With this frequency, we suggest including examination of the periarticular region of the joints in the hands and shoulders whenever a diabetic patient complains of joint pain as many of these complications are potentially treatable, especially if diagnosed early.
